# Zinc utilization and associated factors among under-five children with diarrhea in East Africa: A generalized linear mixed modeling

**DOI:** 10.1371/journal.pone.0243245

**Published:** 2020-12-02

**Authors:** Yigizie Yeshaw, Misganaw Gebrie Worku, Zemenu Tadesse Tessema, Achamyeleh Birhanu Teshale, Getayeneh Antehunegn Tesema

**Affiliations:** 1 Department of Physiology, School of Medicine, College of Medicine and Health Sciences, University of Gondar, Gondar, Ethiopia; 2 Department of Epidemiology and Biostatistics, Institute of Public Health, College of Medicine and Health Sciences, University of Gondar, Gondar, Ethiopia; 3 Department of Human Anatomy, School of Medicine, College of Medicine and Health Sciences, University of Gondar, Gondar, Ethiopia; Universidade de Sao Paulo Faculdade de Saude Publica, BRAZIL

## Abstract

**Introduction:**

Diarrhea is the leading cause of illness and death among under-five children in low and middle income countries. Through the provision of zinc supplements has been shown to reduce the severity and duration of diarrhea, as well as the risk of mortality, the use of zinc for the treatment of diarrhea is still very low in low-income countries. Therefore, this study was conducted to determine the prevalence and associated factors of zinc utilization among under-five children with diarrhea in East Africa.

**Methods:**

A secondary data analysis of the recent Demographic and Health Surveys (DHS) of East African countries were used to determine the prevalence and associated factors of zinc utilization among under-five children with diarrhea in East Africa. A total weighted samples of 16,875 under-five children with diarrhea were included in the study. A generalized linear mixed model (using Poisson regression with robust error variance) was used. Prevalence Ratios (PR) with their 95% confidence interval (CI) were calculated for those variables included in the final model.

**Results:**

The overall prevalence of zinc utilization among under-five children with diarrhea in this study was 21.54% (95% CI = 20.92–22.16). Of East African countries, Uganda had the highest prevalence of zinc utilization (40.51%) whereas Comoros had the lowest (0.44%). Maternal primary education (Adjusted Prevalence Ratio(aPR) = 1.29, 95% CI: 1.16–1.44), secondary education (aPR = 1.36, 95% CI = 1.19–1.55) and higher education (aPR = 1.91, 95% CI = 1.52–2.40), high community women education (aPR = 1.12, 95% CI = 1.02–1.24), high wealth index (aPR = 1.12, 95% CI = 1.01–1.24), high community media exposure (aPR = 1.17, 95% CI = 1.06–1.29) were associated with a higher prevalence of zinc utilization.

**Conclusion:**

The prevalence of zinc utilization among under-five children was found to be low in East Africa. Maternal education, wealth index, community women education, and community media exposure were significantly associated with zinc utilization. Increased mass media exposure, maternal education and wealth index is recommended to improve zinc utilization among under-five children with diarrhea.

## Introduction

Diarrhea is a major cause of morbidity and mortality among under five children worldwide, responsible for an estimated death of 533 768 under 5 children in 2017 [1–3] It is also the leading cause of illness and death among children younger than 5 years in low and middle income countries [4, 5], accounts for an estimated 17.5–21% of all deaths in children under the age 5 years. Of all child deaths from diarrhea, 78% occur in the African and South-East Asian regions, which are also disproportionately burdened with infant and childhood HIV infections [6].

To improve the outcome of diarrhea among under-five children, it is recommended to incorporate zinc into treatment of all young children with diarrhea, and that oral rehydration salts (ORS) [7]. The recommended dosage of zinc for treatment of diarrhea is 20 mg per day for children above six months of age and 10 mg per day for those who are under the age of six months for about 10–14 days [8]. Management of diarrhea through provision of zinc supplements has been shown to reduce the severity and duration of diarrhea, the risk of future episodes within the next two or three months, hospitalization and risk of mortality [9–12]. In addition to this, zinc is important for the reduction of both incidence and prevalence of pneumonia (the leading infectious cause of death among under five children) [13, 14], children growth, improved immune function and brain development, and sexual maturation [15, 16].

Despite, the administration of zinc is one of the most cost effective and affordable way of preventing death among children with diarrhea [17], its global coverage remains extremely low [1]. Nearly all low-income and lower middle-income countries have a policy to use zinc for diarrhea treatment, but the use of zinc for diarrhea is still very low with the exception of a few countries [18]. When we came to sub-Saharan Africa, the proportion of under five children with diarrhea receiving zinc is below 5% [19]. The prevalence of zinc utilization among under five children with diarrhea is 49% in Bangladesh, 18% in Tanzania, 10% in Nigeria, 15% in Sudan [20].

A very limited studies conducted so far identified maternal education [21, 22], economic status [21, 23, 24], mass media exposure [24–28], knowledge about zinc [29, 30] and residence [21, 23, 24] as the factors that are associated with zinc utilization among under-five children with diarrhea.

Despite many advantages of zinc, to the best of our knowledge, there is no study that determines the prevalence and associated factors of zinc utilization among under-five children with diarrhea in East Africa. Therefore, this study was conducted to fill this gap. As this study is the first in determining the magnitude and associated factors of zinc utilization in the region, it can also be used as baseline information for the government and policymakers in taking interventions for the improvement of its utilization.

## Methods

### Study area and data source

This study was conducted in East Africa, the eastern region of the African continent. For this study, the standard DHS survey which is typically collected every five years was used. It is a nationally representative survey that collects data on basic health indicators including zinc utilization among under-five children. The DHS Program used pretested standard Demographic and Health Survey questionnaires to collect data on the population and health issues relevant to each country. The questionnaire was conceptualized to the different countries context and the data were collected by trained data collectors.

The appended kids datasets (KR datasets) of the 9 most recent Demographic and Health Surveys (DHS) of East African countries (Ethiopia 2016, Madagascar 2008, Burundi 2016/17, Kenya 2014, Comoros 2012, Malawi 2015, Tanzania 2015, Uganda 2016, and Zimbabwe 2015) were used to determine the prevalence of zinc utilization and associated factors among under-five children with acute diarrhea in East Africa. Those East African countries with no data on zinc utilization were excluded from the analysis. The DHS surveys of these countries and the detailed data quality control mechanisms of the survey can be found at https://dhsprogram.com/data/dataset_admin/index.cfm. A total weighted sample of 16,875 under-five children with diarrhea in the last two weeks preceding the survey were included.

### Variables of the study

The outcome variable was zinc utilization, which was determined by asking the mother whether zinc is given or not for her child at any time since started diarrhea and then dichotomized as yes if the zinc is given for the child and no otherwise. Since our outcome variable was zinc utilization among children who had diarrhea, only those children who had diarrhea in the last two weeks before the survey were included.

**Fig 1:**
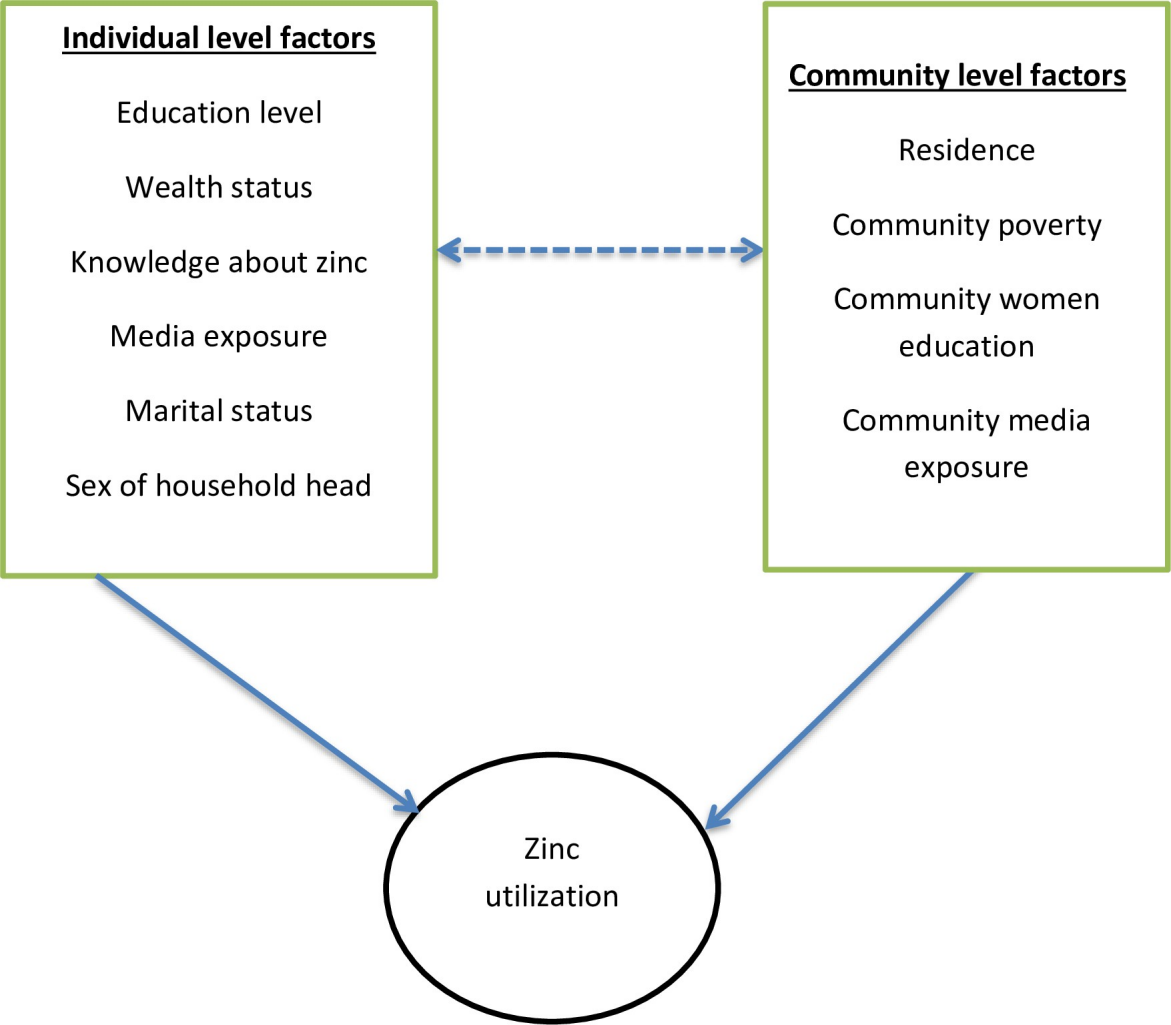
Conceptual framework for zinc utilization as the management of diarrhea among under-five children in East Africa [21–30].

The independent variables for this study include both individual and community level factors. The individual level variables were: marital status(never married, currently married and formerly married), maternal education level(no education, primary education, secondary education and higher education), wealth index(low, middle and high), sex of household head (male or female) and media exposure, a composite variable generated by the aggregation of reading newspaper, listening radio and watching television. Media exposure was dichotomized as yes “if the mother has exposure to either of the above three mentioned media sources” and no “if she doesn’t have exposure to all of the three media sources. The community level variables include: residence (urban or rural), community women education level(aggregate values of community-level maternal education measured by the proportion of women with a minimum of primary level of education derived from data on mothers level of education), community poverty level (proportion of women in the poorest and poorer quintile derived from data on wealth index which is categorized) and community media exposure (proportion of women who had media exposure derived from data on respondents media exposure status (those who had exposure). As stated above, the last three community-level factors were created by aggregating their respective individual level factors at the cluster level (not directly found in DHS) and categorized as high and low based on national median value(their value were not normally distributed) (Fig 1).

### Data analysis procedure

To identify the determinant factors of zinc utilization, STATA 14 software was used. Sampling weight was done before any statistical analysis to adjust for the non-proportional allocation of the sample to different countries and the possible differences in response rates. Since the DHS data has hierarchical nature, measures of community variation/random-effects (Intraclass Correlation Coefficient (ICC), Median Odds Ratio (MOR), and Proportional Change in Variance (PCV)) were estimated. The values of these measures were significant, and hence the use of multilevel model is appropriate. In addition, since the outcome variable was common (21.54%) we used a Poisson regression with robust variance, using generalized linear mixed modeling. Model comparison was done using deviance between the null-model (a model with no independent variable), model I (a model with only individual-level factors), model II (a model with community-level factors) and model III (a model that contain both individual and community level independent variables). A model with the lowest Deviance (model III) was the best fitted model. Both bivariable and multivariable multilevel Poisson regression model was performed to identify the determinant factors of zinc utilization in East Africa. All variables with a p value < 0.25 at bi-variable multilevel Poisson regression analysis were entered into the multivariable multilevel Poisson regression model. P value ≤ 0.05 was used to declare statistically significant variables in the final model.

### Ethics consideration

Since we used a secondary analysis of DHS data, obtaining ethical approval is not necessary. However, to access the datasets, we have registered and received a permission letter to download and use the data files from DHS Program.

## Results

### Sociodemographic characteristics

The total weighted samples of 16,875 under five children with diarrhea were included in this study. The majority, 13,553 (80.32%) of the children were rural. More than half, 8958(53.08%) of the children were males. The majority, 10,805 (64.03%) of their family had media exposure. About half of the participants were from high community media exposure (50.23%) and low community education (50.22%). Eight thousand one hundred and thirty eight (48.23%) of them were from community with high poverty level (Table 1).

**Table 1:** Sociodemographic characteristics of respondents in East Africa.

Variables	Weighted frequency	Percent
Residence		
Urban	3,322	19.68
Rural	13,553	80.32
Sex of child		
Male	8,958	53.08
Female	7,917	46.92
Sex of household head		
Male	12,816	75.95
Female	4,059	24.05
Marital status		
Never married	681	4.04
Currently married	14,432	85.52
Formerly married	1, 762	10.44
Highest maternal education attended		
No education	3,810	22.58
Primary education	9,248	54.80
Secondary education	3,375	20.00
Higher education	442	2.62
Wealth index		
Low	7,988	47.34
Middle	3,232	19.15
High	5,655	33.51
Media exposure		
Yes	10,805	64.03
No	6,070	35.97
Community education level		
Low	8,401	49.78
High	8,474	50.22
Community poverty level		
Low	8,737	51.77
High	8,138	48.23
Community media exposure level		
High	8,476	50.23
Low	8,399	49.77

### Random effect analysis

The results of the random-effects model indicated that there was significant clustering of zinc utilization across the communities (OR of community level variance = 0.046, 95% CI = 0.026–0.081). The ICC in the null model indicated that 1.36% of the overall variability of zinc utilization was attributed to cluster variability. The MOR for zinc utilization was 1.2 in the null model, which indicates that there was a variation in zinc utilization between clusters. This means if we randomly select children from different clusters, children at the cluster with higher zinc utilization had 1.2 times higher prevalence of zinc utilization as compared to those children at cluster with lower zinc use. Model fitness was checked using deviance and the model with the lowest deviance (model III) was the best fitted model (Table 2).

**Table 2:** Model comparison and random effect analysis results.

Parameters	Null model	Model I	Model II	Model III
Community-level variance	0.0455	0.0456	0.0471	0.0470
ICC	1.36%	1.37%	1.41%	1.41%
MOR	1.225	1.225	1.229	1.229
PCV	Ref	0.22%	3.52%	3.30%
Deviance(-2LL)	18052.8804	17973.077	18040.7422	17960.2928

ICC: Intra Class Correlation Coefficient, MOR: Median Odds Ratio, PCV: Proportional Change in Variance.

### Prevalence and determinants of zinc utilization among under-five children with diarrhea

The overall prevalence of zinc utilization among under-five children with diarrhea in this study was 21.54% (95% CI = 20.92–22.16). Of East African countries, Uganda had the highest prevalence of zinc utilization (40.51%) whereas Comoros had the lowest (0.44%) (Table 3).

**Table 3:** Prevalence of zinc utilization among under-five children with diarrhea in East African countries.

Country	Zinc utilization		
	No	Yes	Total number of children with diarrhea
	N (%)	N (%)	
Burundi	2,440(84.95)	432(15.05)	2,872
Ethiopia	807(66.39)	409(33.61)	1,216
Kenya	2,604(91.85)	231(8.15)	2,835
Comoros	489(99.56)	2(0.44)	491
Madagascar	960(98.60)	14(1.40)	974
Malawi	2,545(71.62)	1,008(28.38)	3,554
Tanzania	919(82.44)	196(17.56)	1,115
Uganda	1,675(59.49)	1,140(40.51)	2,815
Zimbabwe	802(79.89)	202(20.11)	1,004

To determine the associated factors of zinc utilization among under-five children with diarrhea, we assessed the crude association between independent variables (residence, sex of household head, marital status, maternal education, wealth index, media exposure, community education level, community poverty level, and community media exposure level) and zinc utilization. Accordingly, only education level, community education, community poverty, wealth index, community media exposure and media exposure were candidate variables for the final model (P<0.25). Then, all of these variables with p-value <0.25 in the bivariable analysis were entered to the multivariable multilevel regression model (final model). Finally, women education, community women education, wealth index and community media exposure were significantly associated with zinc utilization (p≤0.05).

The prevalence of zinc utilization among children from high community education level was 12% higher as compared to children’s from low community education (aPR = 1.12, 95% CI = 1.02–1.24). Compared to children from low wealth index, those children from high wealth index had 12% higher prevalence of zinc utilization (aPR = 1.12, 95% CI = 1.01–1.24). The prevalence of zinc utilization among children with high community media exposure was 17% higher as compared to their counterparts (aPR = 1.17, 95% CI = 1.06–1.29). Compared to children from non-educated mothers, those children born from mothers with primary, secondary and higher education had 29%, 36% and 91% higher prevalence of zinc utilization, respectively (Table 4).

**Table 4:** Multilevel regression analysis of zinc utilization among under-five children with acute diarrhea in East Africa.

	Zinc utilization		Prevalence ratio	
Variables	Yes	No	uPR	aPR
	N (%)	N (%)	(95% CI)	(95% CI)
Education level				
No education	666 (17.49)	3,144 (82.51)	1	1
1^ry^ education	2,026(21.90)	7,222 (78.10)	1.27(1.14–1.40)	1.29(1.16–1.44)*
2^ry^ education	791(23.44)	2,584 (76.56)	1.38(1.22–1.55)	1.36(1.19–1.55)*
Higher education	151 (34.24)	291 (65.76)	1.98(1.60–2.45)	1.91(1.52–2.40)*
Community education				
Low	1,802 (21.45)	6,599 (78.55)	1	1
High	1,832 (21.62)	6,642 (78.38)	1.06(0.97–1.16)	1.12(1.02–1.24)*
Community poverty				
High	1,694 (20.82)	6,444 (79.18)	1	1
Low	1,940 (22.20)	6,797 (77.80)	1.10(1.01–1.19)	1.08(0.98–1.18)
Wealth index				
Poor	1,597 (19.99)	6,391 (80.01)	1	1
Middle	673 (20.83)	2,559 (79.17)	1.05(0.95–1.17)	1.01(0.91–1.13)
High	1,364 (24.12)	4,291(75.88)	1.22(1.12–1.34)	1.12(1.01–1.24)*
Community media exposure				
Low	1,862 (22.18)	6,537 (77.82)	1	1
High	1,772(20.90)	6,704 (79.10)	1.11(1.01–1.21)	1.17(1.06–1.29)*
Media exposure				
Yes	2,400 (22.21)	8,405 (77.79)	1.11(1.02–1.20)	1.03(0.94–1.13)
No	1,234 (20.34)	4,836 (79.66)	1	1

*p≤0.05, uPR: Unadjusted Prevalence Ratio, aPR: Adjusted Prevalence Ratio, CI: Confidence Interval.

## Discussion

This study aimed to determine the prevalence of zinc utilization and associated factors among under-five children with diarrhea in East Africa. The overall prevalence of zinc utilization in this study was 21.54% (95% CI = 20.92–22.16). This magnitude is too far below the global recommendations of zinc utilization [7, 31], which implies that much need to be done to increase its utilization and reduce the impact of diarrhea, preventable cause of under-five mortality in the region [32]. In this study, Uganda had the highest prevalence of zinc utilization compared to other countries in East Africa (40.5%). This might be due the difference in knowledge of women towards diarrhea management following the awareness creation activities for the implementation of a pilot project of improving Infant Young Child Feeding Practices with the optimal use of Micro–Nutrient Powders in different countries. That means the implemented program might have an indirect role for the increment of mothers knowledge, which in turn results in a higher rate of zinc use for the management of diarrhea [29, 30].

The prevalence of zinc utilization in this study is higher than the finding in Tanzania(18%), Nigeria(10%) and Sudan (15%) [31]. The possible reason for this difference is probably due to the differences in sample size in which in the current study; the sample size is too large compared to studies in Tanzania, Nigeria and Sudan. When we simply visualize the figure of the current study with the above studies, it seems there is no as such significant difference. However, when we see the confidence interval in our study, it is too narrow (95% CI = 20.92–22.16) not to include the above findings of previous studies. However, this prevalence is lower than the study in Bangladesh (49%) [31]. This could be again due to socioeconomic and sample size differences between Bangladesh and East Africa region.

In this study, maternal education, wealth index, community women education, and community media exposure were significantly associated with zinc utilization. The prevalence of zinc utilization was higher among children with mothers having primary, secondary and higher education level compared to those children’s of no educated mothers. Similarly, those children who were from high community education level had higher prevalence of zinc utilization compared to children’s of low community education level. This finding is similar with studies in Nigeria [24] and Ethiopia [22]. The plausible possible reason might be those mothers with higher education level usually have an increased awareness and knowledge about the importance of zinc for the management of childhood diarrheal diseases compared to non-educated once [29, 33].

Another factor which is significantly associated with zinc utilization is wealth index. Similar to the findings of studies conducted in Nigeria [24] and Egypt [34], in this study, the prevalence of zinc utilization among children with high wealth index was higher than those children with low wealth index. This is because households who have low wealth index are more likely to encounter budget constraints to buy zinc and use as treatment of diarrhea for their child compared to those who had high wealth index.

In this study, the prevalence of zinc utilization among children with high community media exposure level was higher compared to children from low community media exposure. This finding is similar with the finding in Ghana [26], India [25], and Bangladesh [27]. This is due to the important role of media exposure in increasing caregivers’/mothers knowledge about the management of diarrheal diseases [28].

Our study is not free from limitations. Hence, the following limitations should be considered when interpreting the findings this study. First, the measure of zinc utilization practice was based on mother’s recall which might lead to the possibility of recall bias. Second, the DHS survey does not have data on zinc availability and the contribution of diet to zinc intake. Therefore, we are unable to assess these factors.

Despite the above mentioned limitations, this study used the large representative datasets (Demographic and Health Surveys) of East African countries with multilevel analysis to determine the prevalence and associated factors of zinc utilization among under-five children with diarrhea in East Africa, which is really important to get reliable estimates and will have a vital role for policymakers in taking interventions in the region.

## Conclusion

The prevalence of zinc utilization among under-five children is low in East Africa. Both individual and community level factors were found to be associated with zinc utilization. Higher maternal education, higher wealth index, high community women education, and high community media exposure were significantly associated with higher prevalence of zinc utilization. Increasing media exposure, maternal education and wealth index is recommended to have an increased zinc utilization rate among under-five children with diarrhea in the region.

## List of abbreviations

aPR: Adjusted Prevalence Ratio
CI: Confidence Interval
uPR: Unadjusted Prevalence Ratio
DHS: Demographic and Health Survey
ICC: Intraclass Correlation Coefficient
MOR: Median Odds Ratio
PCV: Proportional Change in Variance
